# ECG alterations suggestive of hyperkalemia in normokalemic versus hyperkalemic patients

**DOI:** 10.1186/s12873-019-0247-0

**Published:** 2019-05-31

**Authors:** Csaba Varga, Zsolt Kálmán, Alíz Szakáll, Kata Drubits, Márton Koch, Róbert Bánhegyi, Tibor Oláh, Éva Pozsgai, Norbert Fülöp, József Betlehem

**Affiliations:** 1 Department of Emergency Medicine, Somogy County Kaposi Mór General Hospital, Tallián Gyula street 20-32, Kaposvár, 7400 Hungary; 2 Institute of Emergency Care and Pedagogy of Health, Faculty of Health Sciences, University of Pécs, Vörösmarty Mihály street 4, Pécs, 7621 Hungary; 3 Hungarian National Ambulance Service, Kossuth Lajos u. 41, Marcali, 8700 Hungary; 4 Department of Oncology, Békés County Kálmán Pándy General Hospital, Semmelweis street 1, Gyula, 5700 Hungary; 5 Department of Surgery, Somogy County Kaposi Mór General Hospital, Tallián Gyula street 20-32, Kaposvár, 7400 Hungary; 6 Institute of Primary Health Care, Medical School, University of Pécs, 7623 Hungary Pécs, Rákóczi street 2, Pécs, Hungary; 7 Institute of Nutritional Sciences and Dietetics, Faculty of Health Sciences, University of Pécs, Vörösmarty Mihály street 4, Pécs, 7621 Hungary

**Keywords:** Hyperkalemia, ECG alterations, Peaked T wave, Wide QRS, Prehospital setting, Periarrest situation

## Abstract

**Background:**

In periarrest situations and during resuscitation it is essential to rule out reversible causes. Hyperkalemia is one of the most common, reversible causes of periarrest situations. Typical electrocardiogram (ECG) alterations may indicate hyperkalemia. The aim of our study was to compare the prevalence of ECG alterations suggestive of hyperkalemia in normokalemic and hyperkalemic patients.

**Methods:**

170 patients with normal potassium (K^+^) levels and 135 patients with moderate (serum K^+^ = 6.0–7.0 mmol/l) or severe (K^+^ > 7.0 mmol/l) hyperkalemia, admitted to the Department of Emergency Medicine at the Somogy County Kaposi Mór General Hospital, were selected for this retrospective, cross-sectional study. ECG obtained upon admission were analyzed by two emergency physicians, independently, blinded to the objectives of the study. Statistical analysis was performed using SPSS22 software. χ^2^ test and Fischer exact tests were applied.

**Results:**

24% of normokalemic patients and 46% of patients with elevated potassium levels had some kind of ECG alteration suggestive of hyperkalemia. Wide QRS (31.6%), peaked T-waves (18.4%), Ist degree AV-block (18.4%) and bradycardia (18.4%) were the most common and significantly more frequent ECG alterations suggestive of hyperkalemia in severely hyperkalemic patients compared with normokalemic patients (8.2, 4.7, 7.1 and 6.5%, respectively). There was no significant difference between the frequency of ECG alterations suggestive of hyperkalemia in normokalemic and moderately hyperkalemic patients. Upon examining ECG alterations not typically associated with hyperkalemia, we found that prolonged QTc was the only ECG alteration which was significantly more prevalent in both patients with moderate (17.5%) and severe hyperkalemia (21.1%) compared to patients with normokalemia (5.3%).

**Conclusions:**

A minority of patients with normal potassium levels may also exhibit ECG alterations considered to be suggestive of hyperkalemia, while more than half of the patients with hyperkalemia do not have ECG alterations suggesting hyperkalemia. These results imply that treatment of hyperkalemia in the prehospital setting should be initiated with caution. Multiple ECG alterations, however, should draw attention to potentially life threatening conditions.

## Background

Hyperkalemia, a relatively common condition in patients, may lead to fatal cardiac arrythmias. In periarrest situations and during resuscitation it is essential to rule out reversible causes, of which hyperkalemia is one of the most common*.* According to the European Resuscitation Council electrolyte disturbances, such as hypo- and hyperkalemia should be corrected preferably before cardiac arrest happens [1]**.** Although, portable devices are readily accessible in prehospital care to measure temperature, oxygen saturation and blood glucose levels, the diagnosis of hyperkalemia in prehospital settings is currently not possible. Since alterations in the ECG-s of patients with hyperkalemia have been documented in a number of investigations, ECG-s have been suggested to facilitate a non-invasive approach to diagnosing hyperkalemia [2]**.**

The concentration of potassium in the serum is tightly regulated between 3.5 and 5.1 mmol/l. As the level of serum potassium increases, typical ECG alterations appear in a characteristic sequence [3]. Mildly elevated potassium levels (5.2–5.9 mmol/l) may cause tall T waves or peaked/tented T waves [3]. Moderately elevated potassium levels (6.0–7.0 mmol/l) typically result in PR interval prolongation, decreased P wave amplitude, disappearance of the P wave, widening of the QRS complex or conduction blocks with escape beats, while severe hyperkalemia (> 7.0 mmol/l) may induce ventricular fibrillation and asystole on the ECG [3].

Despite these ECG manifestations, the clinical diagnosis of hyperkalemia remains difficult as the electrophysiological disturbances listed above are not pathognomic of potassium disorders nor do they appear in each patient with hyperkalemia [4]. According to earlier studies, the prevalence of ECG alterations in hyperkalemia was as low as 14–59% of the cases [4–6]. Reports about severe cases of hyperkalemia with none or minimal ECG alterations have also been published [6, 7].

A number of investigations have studied the correlation between elevated potassium levels and changes in the ECG [4, 5, 8]. To our knowledge, however, no investigation has been conducted regarding the presence of ECG manifestations suggestive of hyperkalemia in patients with normal potassium levels.

The goal of our study was to compare the prevalence of ECG alterations suggestive of hyperkalemia in normokalemic and hyperkalemic patients. It was also our objective to examine the frequency of certain ECG alterations possibly associated with elevated potassium levels. By investigating the frequency of ECG changes in both groups of patients we aimed to elucidate whether these ECG alterations may facilitate recognition of hyperkalemia in the prehospital setting.

## Methods

### Study design

This was an observational retrospective study performed at the Emergency Center of the Kaposi Mór General Hospital in 2013. The study received ethical approval from the regional ethical committee, the Institutional Ethics and Research Ethics Committee of the Somogy County Kaposi Mór Teaching Hospital prior to the research procedure (Reference number: IG/02401–002/2015).

### Patients

The annual census of the Emergency Center is approximately 35,000 patients and 80% of the patients are over 18 years of age. The medical records of adult patients admitted to the Emergency Center between 01.01.2013.-31.12.2013 were chosen randomly from the database of the Emergency Department. We selected 180 patients with normokalemia (3.4–5.1 mmol/l) and 182 patients with moderately (6.0–7.0 mmol/L) or severely (> 7.0 mmol/L) elevated serum or plasma potassium levels, randomly. Patients were required to have had an ECG performed within one hour of the laboratory draw. Patients with hemolysed samples were not included in the study. From the hyperkalemic group, patients with preanalytical errors (15 patients) and patients whose ECG recordings could not be completely analyzed due to technical reasons (16 patients) were excluded as well as patients with multiple presentations (16 cases) within the given interval. Only the first presentation to the Emergency Center was included. In the group with normokalemic patients, 10 patients were excluded due to missing ECG or missing potassium values. Finally, electrocardiograms and data from 135 hyperkalemic (moderate hyperkalemia *n* = 97, severe hyperkalemia *n* = 38) and 170 normokalemic patients were analyzed.

### Laboratory tests

Potassium levels were measured in the Central Laboratory of the Moritz Kaposi General Hospital with Cobas 8000-C702 module according to the protocol of the manufacturer (Roche Diagnostics International AG, Switzerland). The normal range of the potassium level was 3.4–5.1 mmol/l.

### Study protocol

Data including the medical history, comorbidities and medication record were abstracted from the electronical medical record of the hospital. The following data were gathered from each record: demographics (age, gender), serum and plasma potassium levels, ECG, laboratory values (creatinine) obtained on same draw as the potassium level, comorbidities of the patients at the date of admission, medication taken by patients prior to obtaining the ECG, occurance of cardiac arrest. Data was independently reviewed by two investigators.

### ECG analysis

The ECG curves of each patient – recorded within one hour of the blood draw - were analyzed by two board-certified emergency physicians, independently. The physicians were blinded to the objectives and method of the study, to all of the laboratory values (including potassium values), the particular clinical diagnoses and medical histories as well as to each other’s readings. Neither reader was a caregiver for any of the study subjects. Each ECG was examined for the following: rhythm, heart rate, PR interval, AV block, length of QRS interval, ST-T alterations, length of QTc interval. Cardiac arrest was documented in cases of pulseless electrical activity, pulseless ventricular tachycardia, ventricular fibrillation or asystole. PR interval was considered “prolonged” if PR duration> 200 ms, QRS interval was considered “wide” if the QRS duration was > 110 ms. QTc interval was deemed short if QTc < 350 ms and prolonged, if QTc > 450 ms. T waves were considered peaked if they were symmetrical and had a large amplitude based on the investigating physician’s judgement, ECG alterations were considered suggestive of hyperkalemia if the following were recorded: AV junctional escape rhythm, Ventricular escape rhythm, bradycardia, Ist-IInd-IIIrd degree AV blocks, wide QRS, peaked T-waves. Although not generally considered typical ECG manifestations of hyperkalemia, the following ECG changes were also recorded: atrial fibrillation, ST depression, short QTc and prolonged QTc.

### Statistics

Data was analysed using SPSS22 software. In order to test for association of each parameter with measured potassium values, χ^2^ test, or Fischer exact tests were used as appropriate. *P* values ≤0.05 were considered to be statistically significant.

## Results

### Patient characteristics

Data was collected from 135 hyperkalemic (potassium > 6.0 mmol/l) and 170 normokalemic (potassium 3.4–5.9 mmol/l) patients. Gender distribution between the two groups was similar. Patients in the hyperkalemic group were older than patients in the normokalemic (control) group. Almost a third (29.6%) of the hyperkalemic patients suffered from chronic kidney disease (CKD), while only 8.8% of the normokalemic patients had CKD. Accordingly, preceding renal replacement therapy among hyperkalemic patients was significantly more frequent than among normokalemic patients. Regarding medication, a significantly higher percentage of hyperkalemic patients took angiotensin receptor blockers, angiotensin coverting enzyme inhibitors, non steroid analgesics, potassium supplements, and diuretics (including spironolactone, amilorid) than normokalemic patients. Comorbidities, such as heart failure, diabetes mellitus, liver failure, sepsis, cancer and dehydration were also significantly more prevalent in hyperkalemic patients. There was no significant difference between the prevalence of hypertension between normo- and hyperkalemic patients. Mortality within 72 h of admission was high (13.3%) in the hyperkalemic group compared to only 2.4% of normokalemic patients. Data regarding patient characteristics are shown in Table 1.

**Table 1:** Baseline characteristics of normokalemic (control) and hyperkalemic patients

	Age (years)		Female	Prior CKD	Prior RRT	Death within 72 h	need of RRT	HF	DM	HT	Liver failure	Sepsis	Malignancy	Dehydration	B-blocker	Digitalis	ACEi	ARB	Spironolactone	Amilorid	NSAID	K suppl
Kontroll (*n* = 170)	60.45 ± 15.95	%	54,7%	8,8%	1,2%	2,4%	0,0%	11,8%	20,6%	64,1%	4,1%	1,2%	14,1%	10,0%	38,8%	4,1%	37,6%	10,6%	4,7%	1,2%	8,2%	15,3%
		n	93	15	2	4	0	20	35	109	7	2	24	17	66	7	64	18	8	2	14	26
Hyper-kalemia (*n* = 135)	70.61 ± 13.93	%	51,9%	29,6%*	7,4%*	13,3%*	15,6%*	32,6%*	36,3%*	73,3%	11,1%*	14,8%*	29,6%*	31,1%*	45,9%*	5,9%	54,8%*	14,1%*	15,6%*	14,1%*	16,3%*	35,6%*
		n	70	40	10	18	21	44	49	99	15	20	40	42	62	8	74	19	21	19	22	48

(CKD: chronic kidney disease, RRT renal replacment therapy, HF: heart failure, DM: diabetes mellitus, HT: hypertension, K suppl: potassium supplementation, *: *p* ≤ 0,05)

### The frequency of ECG alterations suggestive of hyperkalemia in normokalemic versus hyperkalemic patients

The frequencies of ECG alterations suggestive of hyperkalemia were recorded in patients with normokalemia, moderate hyperkalemia and severe hyperkalemia.

**Fig. 1:**
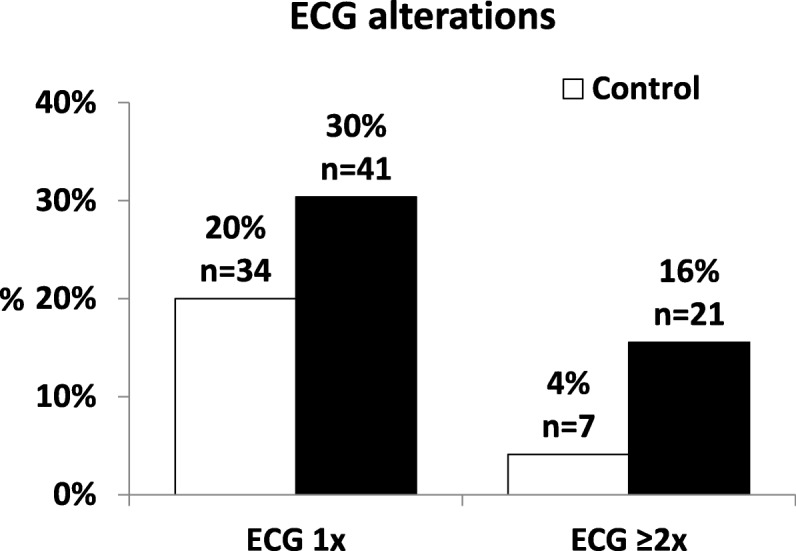
The frequency of single and multiple ECG alterations suggestive of hyperkalemia in normokalemic and hyperkalemic patients

In the control group, 24.0% of normokalemic patients had ECG alterations suggestive of hyperkalemia and from these, 20% had one and 4% had 2 or more ECG changes indicative of hyperkalemia. Less than half of the patients (46%) with moderate or severe hyperkalemia had some form of ECG manifestation suggestive of hyperkalemia. 29% of severely hyperkalemic patients had no ECG changes indicative of hyperkalemia. From the 46% of hyperkalemic patients exhibiting some form of hyperkalemic ECG manifestation, 30% had one and 16% had two or more ECG alterations suggesting hyperkalemia. (Table 2, Fig. 1).

**Table 2:** Frequency of ECG alterations suggestive of hyperkalemia in normokalemic versus hyperkalemic (moderate and/or severe) patients (*: *p* ≤ 0,05), Cardiac arrest included asytole, ventricular fibrillation, pulseless ventricular tachycradia and pulseless electric activity

		1 ECG alteration	≥2 ECG alterations	Cardiac arrest	AV junctional escape	Ventricular escape	Bradycardia	I°degree AV-block	II°degree AV-block	III°degree AV-block	Wide QRS	Peaked T-waves
Control (n = 170)	%	20%	4%	0.0%	0.0%	0.0%	6.5%	7.1%	0.0%	0.0%	8.2%	4.7%
	n	34	7	0	0	0	11	12	0	0	14	8
All hyperkalemia (n = 135)	%	30%	16%	5.9%	5.2%	0.7%	12.6%	8.1%	0.0%	0.7%	18.5%*	9.6%
	n	41	21	8	7	1	17	11	0	1	25	13
Moderate hyperkalemia (n = 97)	%	27%	9%	3.1%	5.2%	1.0%	10.3%	4.1%	0.0%	0.0%	13.4%	6.2%
	n	26	9	3	5	1	10	4	0	0	13	6
Severe hyperkalemia (n = 38)	%	39%	32%	13.2%	5.3%	0.0%	18.4%*	18.4%*	0.0%	2.6%	31.6%*	18.4%*
	n	15	12	5	2	0	7	7	0	1	12	7

**Fig. 2:**
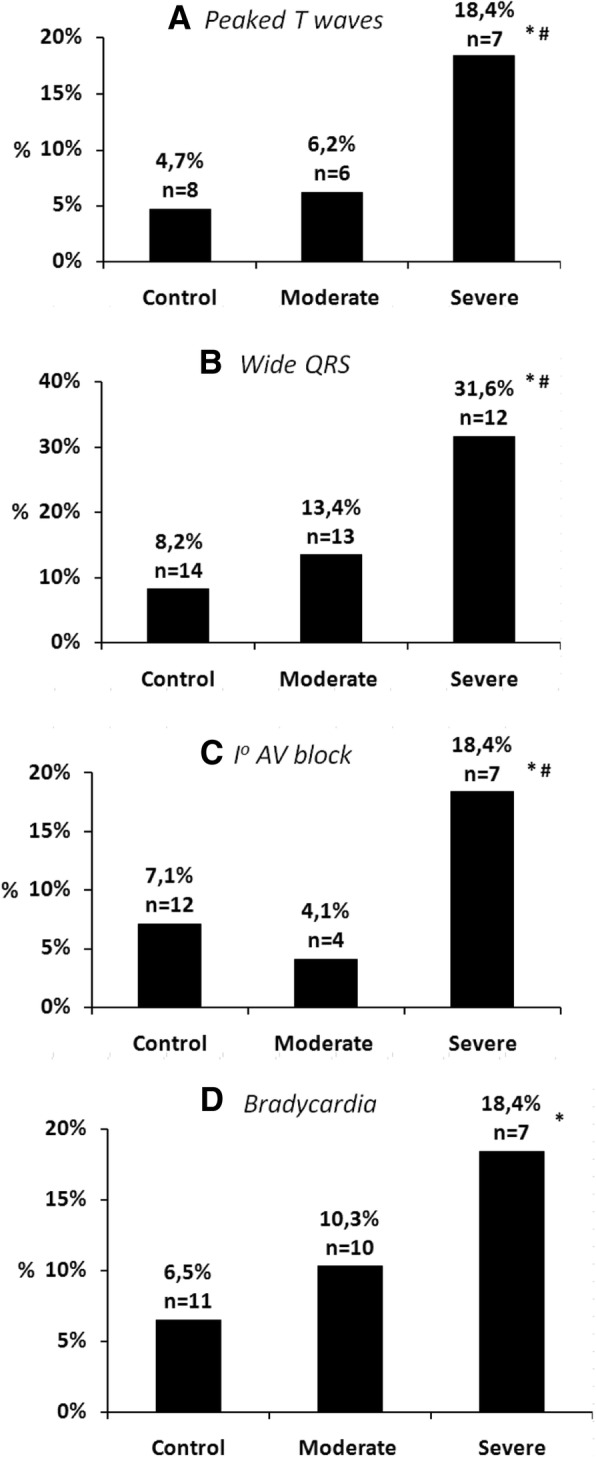
The frequency of ECG alterations suggestive of hyperkalemia in normokalemic, moderately hyperkalemic and severely hyperkalemic patients (*: *p* < 0.05 vs control, #: *p* < 0.05 vs moderate hyperalemia) **a**: Peaked T waves; **b**: Wide QRS; **c**: Ist degree AV block; **d**: Bradycardia

Significantly more patients with severe hyperkalemia had wide QRS (31.6%), bradycardia (18.4%), peaked T-waves (18.4%) and 1st degree AV block (18.4%) compared to normokalemic patients (8.2, 6.5, 4.7, and 7.1%, respectively). Wide QRS (31.6%) was the most common ECG alteration in severely hyperkalemic patients and in the normokalemic group as well. (Table 2*,* Fig. 2.) There was no significant difference in the frequency of ECG alterations suggestive of hyperkalemia between the normokalemic and the moderately hyperkalemic groups. (Table 2, Fig. 2).

When normokalemic patients were compared to all (moderately + severely) hyperkalemic patients, we found that wide QRS (18.5%) was the only ECG alteration significantly more frequent in all hyperkalemic patients compared to normokalemic patients (8.2%). AV junctional rhythm was seen only on the ECG of hyperkalemic (moderate or severe) patients but was not present on ECG recorded for normokalemic patients. (Table 2*,* Fig. 2).

Cardiac arrest occurred in 8 patients with hyperkalemia and 5 of these patients had severely high potassium levels. None of the patients with normal potassium levels had cardiac arrest. (Table 2).

### The frequency of ECG alterations possibly associated with hyperkalemia in normokalemic versus hyperkalemic patients

Frequency of ECG abnormalities possibly associated with hyperkalemia including atrial fibrillation, QTc changes and ST depression were registered.

There was no significant difference regarding the frequency of ST depression between patients with normal or increased levels of potassium. Atrial fibrillation was significantly more frequent in severely hyperkalemic patients (26.3%) compared to normokalemic patients (10.6%). However, no significant difference regarding atrial fibrillation was detected between the moderately hyperkalemic and the normokalemic groups. Prolonged QTc was the only ECG alteration which was significantly more prevalent in both patients with moderate (17.5%) and severe hyperkalemia (21.1%) compared to patients with normokalemia (5.3%). (Table 3*,* Fig. 2).

**Table 3:** The frequency of ECG alterations possibly associated with hyperkalemia in normokalemic versus hyperkalemic patients. (*: p ≤ 0.05)

		AF	ST depression	Short QTc	Prolonged QTc
Control (n = 170)	%	10.6%	28.8%	0.0%	5.3%
	n	18	49	0	9
All hyperkalemia (n = 135)	%	15.6%	23.0%	0.7%	18.5%*
	n	21	31	1	25
Moderate hyperkalemia (n = 97)	%	11.3%	22.7%	1.0%	17.5%*
	n	11	22	1	17
Severe hyperkalemia (n = 38)	%	26.3%*	23.7%	0.0%	21.1%*
	n	10	9	0	8

### The association between the occurance of certain ECG alterations and the severity of hyperkalemia

**Fig. 3:**
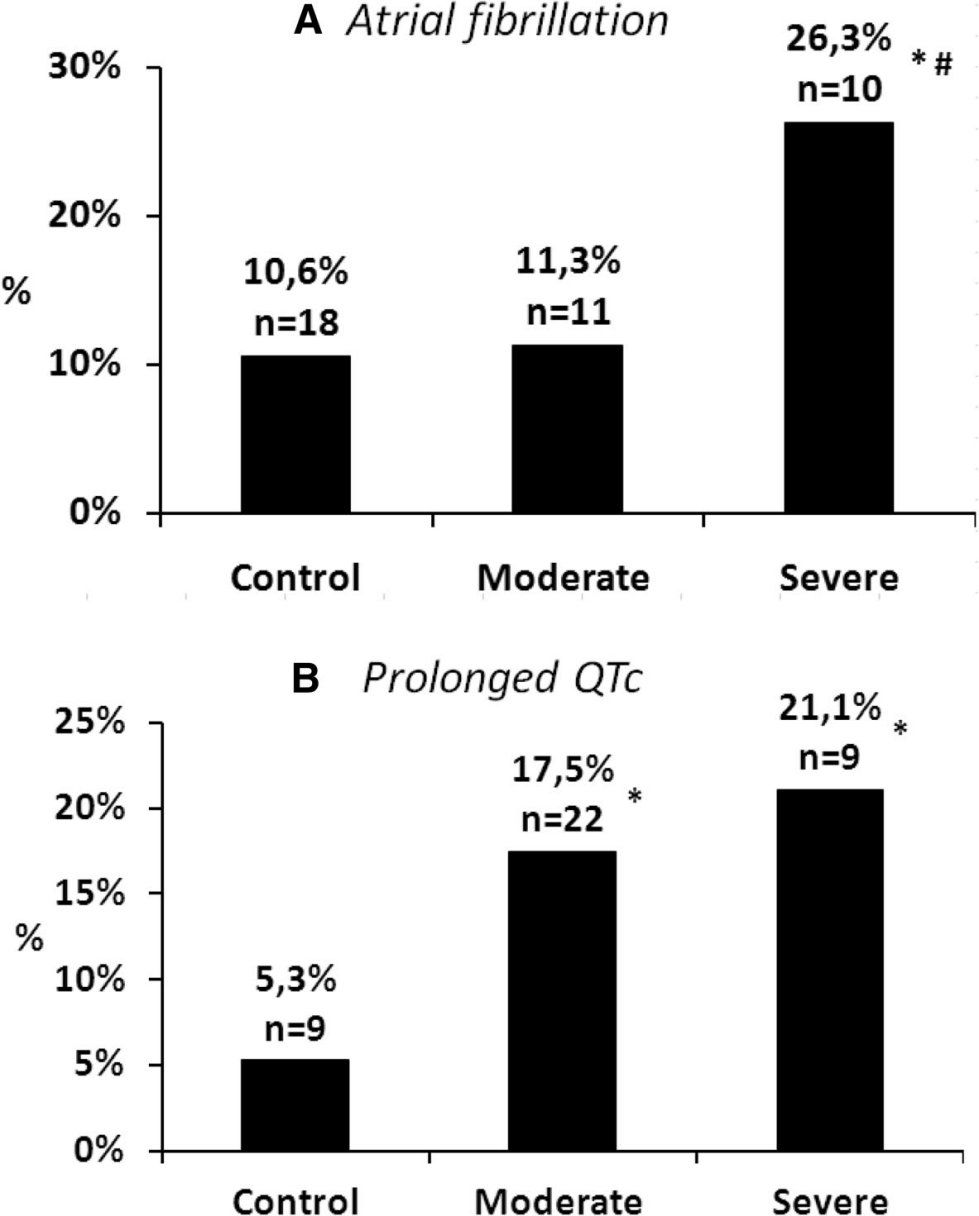
The frequency of ECG alterations possibly associated with hyperkalemia in normokalemic versus hyperkalemic patients

We investigated whether the elevations of potassium levels increased the occurrence of certain ECG alterations. The following differences were found between moderately and severely hyperkalemic patients: atrial fibrillation (11.3 vs 26.3%, χ^2^ test, *p* < 0.05), I^o^ AV block (4.1% vs 18.4%, Fisher exact test, p < 0.05,) wide QRS (13.4% vs 31.6%, χ^2^ test, p < 0.05) and peaked T waves (6.2% vs 18.4%, Fisher exact test, *p* = 0.05) were present more frequently on the ECG recordings of patients with severe hyperkalemia than on the ECG of patients with moderate hyperkalemia. (Figs. 2 and 3).

## Discussion

Hyperkalemia is one of the most common, potentially life-threatening metabolic disorders of reversible periarrest conditions that needs to be recognized and treated in time [9]. Potassium levels above the normal range often remain unnoticed and periarrest situations may occur without warning [10]. Electrocardiography is a widely used, easily attainable method to raise the possibility of hyperkalemia, however there have been conflicting reports about its sensitivity and specificity to signal elevated potassium levels [3, 5, 11].

In our study we investigated whether ECG alterations suggestive of hyperkalemia were present in a randomly selected group of normokalemic patients admitted to the Emergency Center. Previous studies have shown that the ability of physicians to predict hyperkalemia from the ECG was low with sensitivities between 0.43 and 0.34 and experienced readers’ ability to predict severity of hyperkalemia were likewise poor [5, 11]**.** ECGs have often been shown to be normal in hyperkalemia and a number of cases have been reported where patients with severely elevated potassium levels did not show typical ECG manifestations [7, 11]. In accordance with these investigations, we found that less than half of the hyperkalemic patients exhibited ECG changes suggestive of hyperkalemia, while the majority of the hyperkalemic patients showed no typical ECG changes at all. A surprisingly high proportion (24%) of normokalemic patients exhibited ECG alterations suggestive of hyperkalemia. Thus, based on ECG analysis alone, normokalemia and hyperkalemia cannot be confirmed or exluded in patients.

It must be noted, however, that some ECG changes suggestive of hyperkalemia (wide QRS, peaked-T waves, 1st degree AV-block and bradycardia) were significantly more prevalent in the severely hyperkalemic group. Although there wasn’t a significant difference between the frequency of ECG alterations suggesting hyperkalemia in normokalemic and moderately hyperkalemic patients, the number of ECG alterations suggestive of hyperkalemia simultaneously present increased with the degree of serum potassium elevation. These findings are in line with a previous report on the higher frequency of ECG changes with increasing potassium levels [4]. Peaked T waves are considered to be the typical earliest ECG signs of elevated serum potassium levels [3–5]**.** In our study, peaked T-waves was the second most common ECG manifestation among severely hyperkalemic patients, while wide QRS was the most common ECG change and significantly more often found among all hyperkalemic patients compared to patients with normal potassium levels.

We examined the prevalence of four ECG abnormalities, whose association with hyperkalemia has been found to be equivocal according to previous studies**.** ST depression may be an ECG manifestation of hyperkalemia [12], but we did not detect a significant difference in the frequency of ST depression between normokalemic and hyperkalemic patients in our study. Atrial fibrillation has been associated with certain changes in the ECG and most studies have found that lower potassium levels were associated with a higher risk of atrial fibrillation [13–15]**.** Our results showed, however, that atrial fibrillation was more prevalent in severely hyperkalemic patients compared to normokalemic patients. We attribute these results to the synergistic effect of two groups of diseases often present in patients with high potassium levels. Hyperkalemia and heart failure are common in chronic kidney disease and heart failure is often the cause of or caused by atrial fibrillation. Therefore, atrial fibrillation occurs not as the result of hyperkalemia but rather as the consequence of illnesses often associated with hyperkalemia.

Although shortening of the QTc interval in hyperkalemia has been reported in several investigations [12, 16], the possibility of prolonged QTc occurance in hyperkalemia has also been raised [17]**.** In our study, prolonged QTc were more frequent in severely hyperkalemic patients compared to normokalemic patients. In fact, prolonged QTc was the only ECG alteration significantly more frequent in both moderately and severely hyperkalemic patients compared to the group with normal potassium levels. Although the reasons underlying our findings need to be clarified, the results imply that prolonged QTc and atrial fibrillation could also draw attention to hyperkalemia, besides the more acknowledged ECG manifestations of hyperkalemia.

Our study has other implications. When we compared baseline data from patients in the normokalemic and hyperkalemic groups, CKD and comborbidities such as heart and liver failure were significantly more frequent in hyperkalemic patients. CKD and/or hemodialysis have been known to increase the likelihood of hyperkalemia [4, 18] and so patients suffering from renal failure are more at risk of developing potassium cardiotoxicity [19]. The usage of certain types of medication, including ACEI, ARB and potassium-sparing diuretics has been associated with an increased number of hyperkalemia-related hospitalizations and mortality [20–23]. In keeping with earlier studies, we found that the application of these types of drugs was also more common in patients with elevated potassium levels. Our data underline the importance of regular monitoring of electrolytes in patients taking hyperkalemia-inducing medication, preferably already in non-urgent situations within the primary care setting since ECG diagnosis of hyperkalemia is uncertain.

To our knowledge, this study is the first to investigate ECG alterations suggestive of hyperkalemia in a large number of patients with normal potassium levels. Besides supporting evidence for the unreliability of ECG diagnosis in hyperkalemia, our results show that a fourth of normokalemic patients also exhibit ECG alterations suggestive of hyperkalemia. This indicates that treatment of suspected hyperkalemia in the prehospital setting, prior to laboratory confirmation of potassium levels, may not be prudent. Although it has been suggested that initiation of life-saving treatment with calcium in suspected hyperkalemia prior to laboratory confirmation of hyperkalemia would be advisable, we disagree with this proposition [24]. Investigations have shown, that the empiric treatment of hyperkalemia based on ECG alone was predicted to lead to the mistreatment of at least 15% of patients [11] and treatment decisions should not depend only on the presence of ECG alterations [4].

### Limitations

Our study has several limitations. The normokalemic (control) group and hyperkalemic group were not matched regarding age, medication and comorbidites. This was to be expected, however, as this investigation was a cross-sectional study of patients admitted to emergency care. The interpretation of ECGs may be another confounding factor, since a number of ECG alterations can be due to other causes than hyperkalemia or alternatively, ECG changes due to other conditions may mask signs of hyperkalemia. We argue, however, that in the prehospital, emergency setting information regarding medication and previous illnesses is not readily available to the caregiver and therefore, he or she must make decisions regarding diagnosis and treatment with none or very limited information. Another limitation of our study is that although the investigation was conducted on a relatively large number of patients, a larger-scale study examining the ECG alterations suggestive of hyperkalemia in normokalemic patients would be needed to confirm our results.

## Conclusion

Our findings imply, what previous studies have suggested, that the ECG is not a reliable tool in the diagnosis of hyperkalemia. To this, our study adds valuable information by demonstrating that a minority of patients with normal potassium levels may also exhibit ECG alterations considered to be typical for hyperkalemia. These results underline the importance of accurate laboratory-confirmed diagnosis of hyperkalemia prior to initiation of treatment. Nevertheless, since ECG alterations suggestive of hyperkalemia are more frequent in hyperkalemic patients, any change in the ECG attributable to hyperkalemia should draw attention to a potentially life threatening condition, in the prehospital emergency setting, especially in periarrest situations.

## Data Availability

The datasets used and/or analysed during the current study are available from the corresponding author on reasonable request.

## Abbreviations

CKD: Chronic kidney disease
DM: Diabetes mellitus
ECG: Electrocardiogram
HF: Heart failure
HT: Hypertension
K suppl: Potassium supplementation
RRT: Renal replacement therapy
